# Comparison of three-dimensional imaging of the nose using three different 3D-photography systems: an observational study

**DOI:** 10.1186/s13005-024-00406-4

**Published:** 2024-01-24

**Authors:** Lucas M. Ritschl, Carolina Classen, Paul Kilbertus, Julia Eufinger, Katharina Storck, Andreas M. Fichter, Klaus-Dietrich Wolff, Florian D. Grill

**Affiliations:** 1 Department of Oral and Maxillofacial Surgery, School of Medicine and Health, Technical University of Munich, Klinikum rechts der Isar, Ismaninger Strasse 22, D-81675 Munich, Germany; 2 Department of Oral and Maxillofacial Surgery, Saarland University Medical Centre, 66421 Homburg, Germany; 3 Department of Otorhinolaryngology, Head and Neck Surgery, School of Medicine and Health, Technical University of Munich, Klinikum rechts der Isar, Ismaninger Strasse 22, D-81675 Munich, Germany; 4 Private Practice Oral and Maxillofacial Surgery, Wolfratshausen, Germany

**Keywords:** 3D photography, Rhinoplasty, 3D technologies, Face scan, TrueDepth

## Abstract

**Background:**

New 3D technologies for superficial soft tissue changes, especially in plastic and reconstructive surgical procedures, can improve the planning and documentation of facial surgeries. The purpose of this study was to compare and determine the applicability and feasibility of three different 3D-photography systems in clinical practice imaging the nose.

**Methods:**

A total of 16 healthy non-operated noses were included in this prospective study. A plaster model of each nose was produced, digitized, and converted to a .stl mesh (= ground truth model). Three-dimensional images of each nose were then taken using Artec Space Spider (gold standard), Planmeca ProFace®, and the Bellus3D Dental Pro application. All resulting .stl files were aligned to the ground truth model using MeshLab software, and the root mean square error (RMSE), mean surface distance (MSD), and Hausdorff distance (HD) were calculated.

**Results:**

The Artec Space Spider 3D-photography system showed significantly better results compared to the two other systems in regard to RMSE, MSD, and HD (each *p* < 0.001). There was no significant difference between Planmeca ProFace® and Bellus3D Dental Pro in terms of RMSE, MSD, and HD. Overall, all three camera systems showed a clinically acceptable deviation to the reference model (range: -1.23–1.57 mm).

**Conclusions:**

The three evaluated 3D-photography systems were suitable for nose imaging in the clinical routine. While Artec Space Spider showed the highest accuracy, the Bellus3D Dental Pro app may be the most feasible option for everyday clinical use due to its portability, ease of use, and low cost. This study presents three different systems, allowing readers to extrapolate to other systems when planning to introduce 3D photography in the clinical routine.

## Background

Advances in three-dimensional (3D) technologies have revolutionized the field of plastic and reconstructive surgery, providing surgeons with new tools to plan and execute procedures with unprecedented accuracy and precision. In particular, the use of 3D photography has gained popularity in recent years, offering a non-invasive and objective way to capture preoperative anatomical details of the face and body [1, 2].

Three-dimensional photography involves the use of specialized cameras and software to create a 3D digital surface model of the subject [3]. These models can be used to simulate surgical outcomes, aiding in preoperative planning, communication between surgeon and patient, and postoperative evaluation. Three-dimensional photography can also serve as a valuable tool in documentation, teaching and research, enabling objective measurements and comparisons of surgical results [4].

In the context of facial plastic surgery, 3D photography has been particularly useful for rhinoplasty, a complex surgical procedure that involves reshaping the nose for functional and aesthetic purposes. Its use in rhinoplasty allows surgeons to capture the precise anatomical details of the nose, including the shape, size, and symmetry of the nasal structures [5]. With the information obtained, the rhinoplasty can first be digitally simulated, planned and evaluated with the patient’s expectations. Postoperatively, the facial swelling can be objectively monitored with the help of 3D photography. In orthognathic surgery, 3D photography also offers valuable advantages in treatment planning and outcome assessment, by improving the soft-tissue simulation of the bony repositioning [6]. By capturing a detailed 3D digital model of the patient’s face and jaw, surgeons can accurately analyze the relationship between the skeletal structures, soft tissues, and dental occlusion [7]. This information aids in the precise planning of surgical movements and helps optimize the aesthetic and functional outcomes of the procedure. Furthermore, 3D photography can be used for progress monitoring for patients with cleft lip and palate and craniosynostoses. Despite the potential benefits of 3D photography, there are several challenges associated with its implementation in clinical practice. The accuracy and reliability of 3D-photography systems vary, and the cost and lack of portability of these systems can limit their widespread adoption [8]. Additionally, the interpretation and use of 3D data requires specialized training and expertise [9].

This study aims to compare and determine the applicability and feasibility of three different 3D-photography systems in clinical practice for imaging the complex geometry of the nose with regard to accuracy, precision, feasibility, and cost-effectiveness.

## Methods

### Ethical statement and patient recruitment

All clinical investigations and procedures were conducted according to the principles expressed in the Declaration of Helsinki. The written informed consent was obtained from all participants. This study was approved by the Ethical Committee of the Technische Universität München (Approval No. 240/21 S-EB).

Sixteen healthy participants who met the inclusion criteria (voluntary participation, age of majority, no history of nasal surgery or other nasal abnormalities) between January 2020 and December 2020 at the department of Oral and Maxillofacial Surgery, School of Medicine and Health, Technical University of Munich, Klinikum rechts der Isar were included.

### Workflow and surface-based comparison, and alignment

**Fig. 1:**
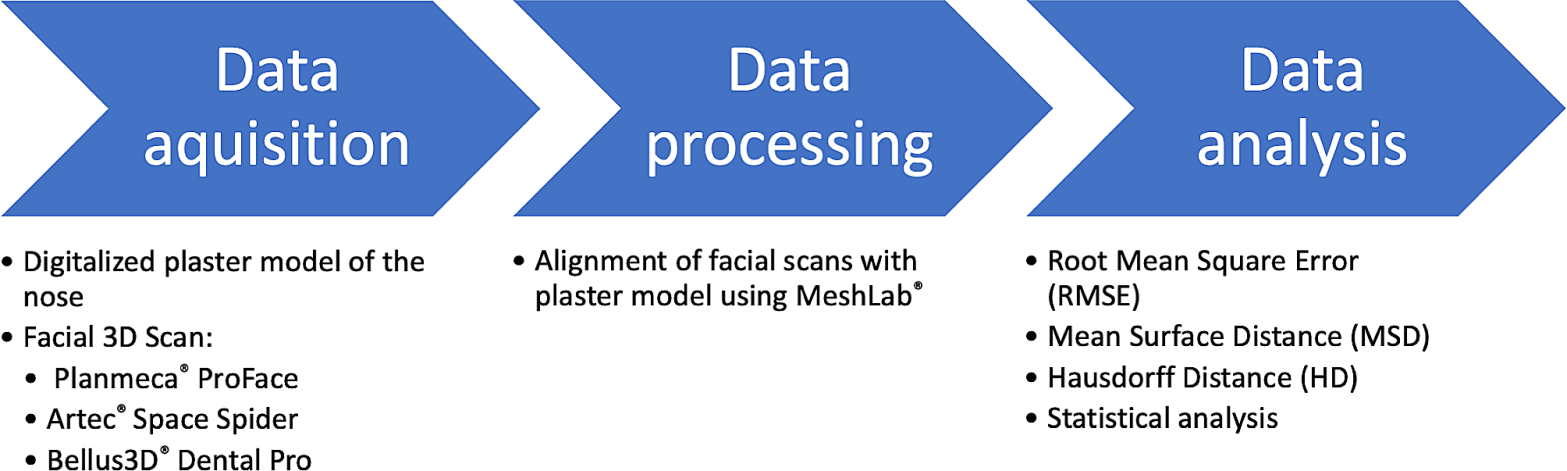
Workflow for comparison of three-dimensional imaging of the nose using three different 3D-photography systems: data acquisition – data processing – data analysis

The workflow for this study involved three steps: data acquisition, processing, and analysis (Fig. 1). Data acquisition involved creating a ground truth model of the nose using a conventional impression of the nose and fabricating a plaster model of the nose. An A-silicone Memosil® 2 (Hereaeus Kulzer GmbH, Hanau, Germany) was used as impression material because of its medium viscosity, which allows for an accurate impression, and its transparency, which ensures visual control during the impression-taking process [10]. Once the silicone impression was taken, a plaster model of the nose was fabricated. The plaster model was then digitized into an .stl file format using the 3Shape D500 scanner (3Shape® A/S, Denmark). The 3Shape D500 scanner is a high-precision dental laser scanner that is designed for capturing high-resolution scans with an accuracy of 20 μm. The scanner is equipped with two built-in 1.3-megapixel cameras and a three-axis joint rotation system, which allows for easy positioning of the model during scanning [10]. Overall, the combination of the A-silicone Memosil® 2 and the 3Shape D500 scanner allowed for the creation of a highly accurate and detailed ground truth model of each nose.

**Fig. 2:**
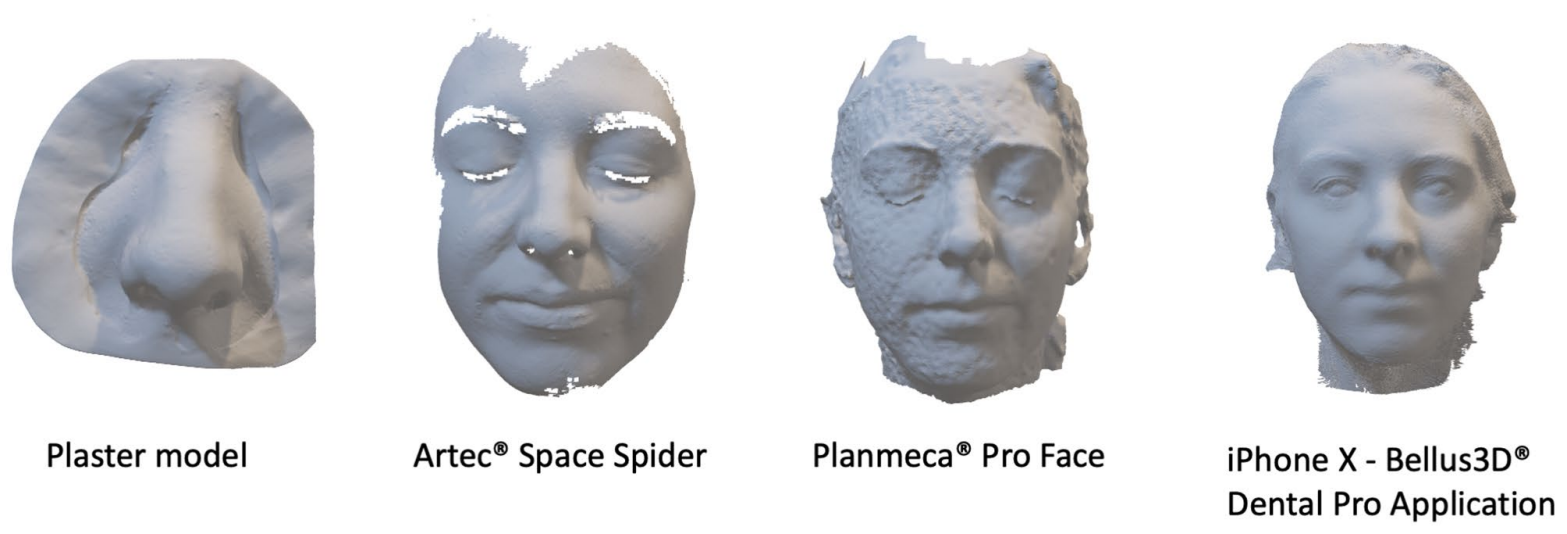
Data acquisition using three different 3D-photography systems

**Fig. 3:**
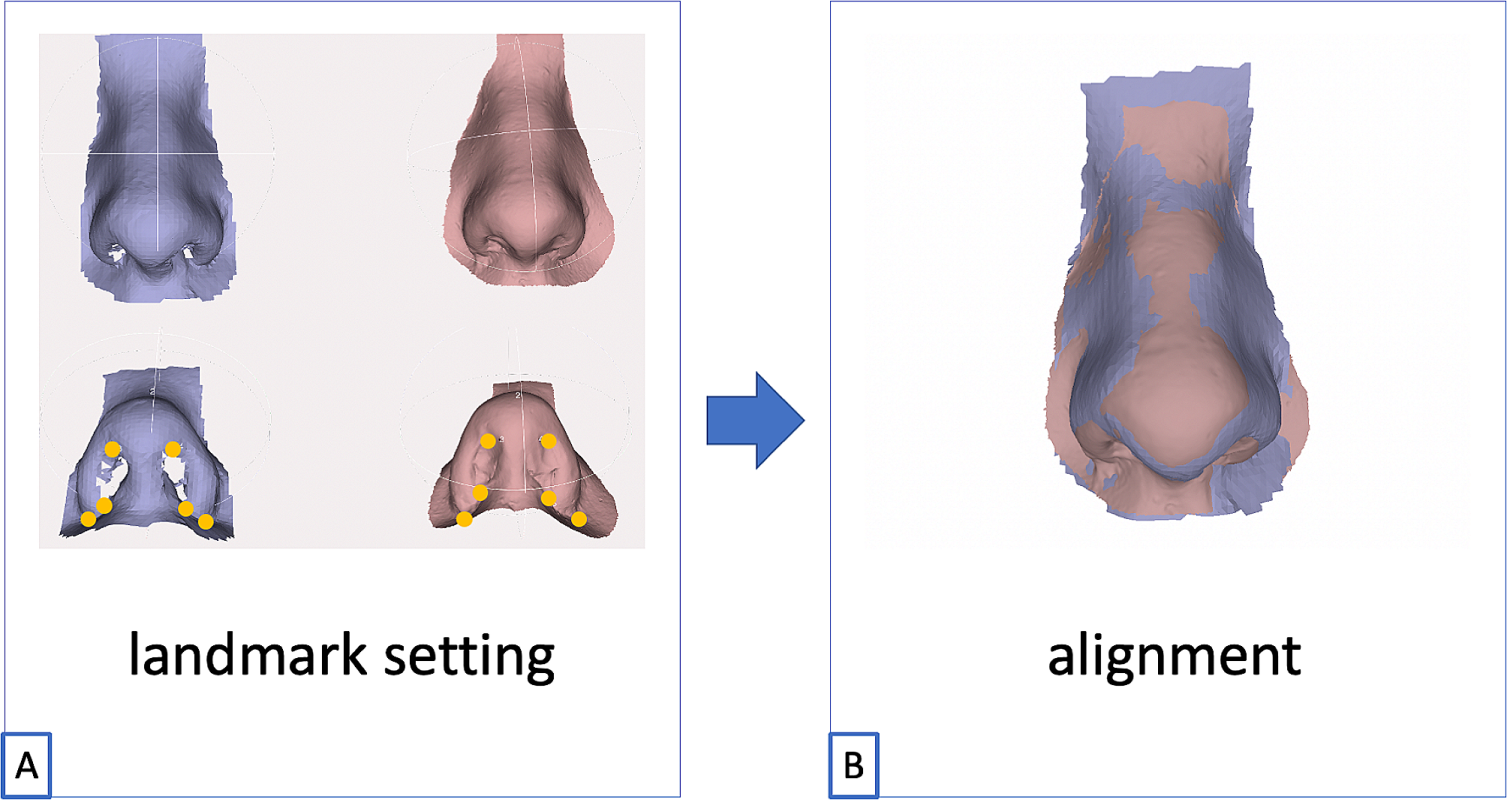
**A:** Alignment and overlay of the plaster model to the nose acquired with Artec Space Spider in MeshLab® using the ICP algorithm Purple: .stl dataset of the nose to be superimposed; Beige: reference model Yellow dots/landmarks: Nostril top right/left, nostril base point right/left, alare right/left **B:** Both models after semi-automatic superposition

In the next step, 3D surface models of the corresponding noses were captured with three different 3D-photography systems (see below). The resulting geometries were also saved as .stl files (Fig. 2). Data processing involved aligning the captured corresponding surfaces with the ground truth model using MeshLab software (Version 2021.05d; Pisa, Italy). The Iterative Closest Point (ICP) algorithm in MeshLab was used to align the captured images with the corresponding ground truth model [11, 12]. The mathematical algorithm is based on bringing two-point clouds via rotations and translations into congruence as far as possible [11] (Fig. 3). Therefore, defined landmarks taken from the color atlas *Three-Dimensional Cephalometry* by Gwen R.J. Swennen were used in this study to initiate the algorithm [13]. This pairwise local alignment was then applied to a set of ground truth model plus another .stl mesh, bringing them closer to each other. The software then automatically selects corresponding points between the two sets, and the algorithm iteratively refines the global alignment until the error between the two sets is minimized. The original alignment code used in Meshlab is a derivative of the one used in Scanning Tools of the Visual ComputingLab published by Callieri et al. [14]. After alignment, the root mean square error (RMSE), mean surface distance (MSD), and Hausdorff distance (HD) were calculated in MeshLab to evaluate the accuracy and precision.

In addition to the calculated parameters, we created surface distance maps. These were used to visualize the differences between the two models and help to identify areas where the imaging system may have inaccuracies or limitations. Areas with a small distance are displayed in our study in green, while areas with a larger distance are displayed in blue.

All measurements were performed independently by two investigators (CC and PK). All analyses were performed twice; the second round of analysis was performed seven to fourteen days later to minimize a habitual landmark setting that initiates the ICP algorithm [15].

### 3D-photography systems

Artec Space Spider is a high-resolution 3D scanner designed for use in manufacturing for quality control, reverse engineering, and prototyping applications, capable of capturing details with an accuracy of up to 0.05 mm [16]. Artec Space Spider utilizes blue LED structured light scanning technology to capture high-resolution 3D scans of objects with a scan speed of up to 7.5 frames per second [16]. The acquisition costs for Artec Space Spider amount to approximately €20,000 excluding laptop. Added to this is approximately €2,000 a year for the annual software license.

Planmeca ProFace® is a 3D facial scanning system designed to capture a realistic 3D face photo and a cone beam computed tomography (CBCT) image with a single scan. The 3D face photo can also be created separately without exposing the patient to any radiation, as in this study. According to the manufacturer, this is a passive stereophotogrammetry system with two integrated stereo cameras. The cameras each have a resolution of 1928 × 1088 megapixels. The 3D image is created by stitching together the different surfaces reconstructed from the stereo images of different views. In addition to the acquisition costs of the CBCT, the ProFace® package from Planmeca costs approximately €4,000.

Bellus3D Dental Pro was a mobile application, which was used as a representative for the *iPhone X TrueDepth system and is* designed for dental professionals to capture 3D scans of a patient’s face using an iPhone or iPad with the TrueDepth camera [17]. According to the manufacturer, the application reconstructs the 3D dataset from seven different individual images. The TrueDepth camera of the iPhone captures the facial surface by projecting 30,000 invisible dots onto the face using a “dot projector” and analyzing them with the help of the infrared camera. Color is superimposed on each dot by integrating the 7-megapixel camera [17]. The application was originally designed to be used by dental professionals for a variety of purposes, including treatment planning, orthodontic assessment, and implant placement. In addition to its dental applications, the Bellus3D Dental Pro application can also be used for cosmetic and reconstructive facial surgery planning. The high-resolution 3D scans can provide detailed information about the patient’s facial structure, which can help the surgeon to plan the surgery and achieve optimal results. This application is limited to Apple devices equipped with the TrueDepth camera. The monthly cost of the application is €40 excluding the purchase cost of the iPhone.

### Statistical analysis

Statistical analysis was performed using IBM SPSS 24 for Mac software (IBM Corp, Armonk; New York, United States). The intraclass correlation coefficient (ICC) was calculated to determine the intra- and interrater reliability and consistency of measurements performed by two raters applying a two-way mixed model. For the differences of RMSE, MSD, and HD, one-factor analysis of variance to test for significant differences between the three 3D-photography systems (Artec Space Spider, Planmeca ProFace®, and Bellus3D Dental Pro) was performed. Univariate linear regression analysis was performed to analyze possible confounding factors on RMSE, MSD, and HD. All statistical tests were performed on an exploratory two-sided 5% significance level. No adjustments were made for multiple testing.

## Results

### General parameters

General information of the enrolled study population is shown in Table 1. The study population consisted of a total of 16 participants. Of the volunteers, nine were male and seven were female. The median age at the time of the study was 29.8 years (range 20–36 years). For all subjects, all 3D photographs and the digitized plaster model could be analyzed, so we were able to analyze a total of 64 3D datasets in .stl format.

**Table 1:** Overview and demographics of enrolled patients regarding registered parameters: gender, age, ethnicity, number of 3D .stl files

Parameters	n (%)	
Gender female/male	7/9	
Age median (range)	29.8 (20–36)	
Ethnicity	`Caucasian	15 (93.75%)
	Asian	1 (6.25%)
Number of captured 3D .stl files	Plaster casts	16 (100%)
	Planmeca® ProFace	16 (100%)
	Artec® Space Spider	16 100%)
	Bellus3D® Dental Pro	16 (100%)

### The intraclass correlation coefficients

To analyze the interrater reliability of the two independent investigators, the intraclass correlation coefficient (ICC) was calculated based on the measurement performed using a mixed two-way model (Table 2). The absolute values of the individual measurements were used for the calculation. At the same time, this analysis also served to assess precision as a measure of the agreement between independently determined measured values. The measurements showed that there is a very good (ICC > 0.9) interrater reliability for all three parameters and for all three 3D-photography systems. The ICC was also used to analyze the intrarater reliability (Table 3). The measurements also showed consistently good (ICC = 0.81–0.9) to very good intrarater reliability (ICC > 0.9) for both investigators.

**Table 2:** Intraclass correlation (ICC) to analyze the *interrater* reliability of measurements performed by the two independent raters applying a two-way mixed model

Parameter	ICC	95% CI
RMSE Planmeca® ProFace	0.999	0.997–0.999
RMSE Artec® Space Spider	0.994	0.987–0.997
RMSE Bellus3D® Dental Pro	0.996	0.991–0.998
MSD Planmeca® ProFace	0.923	0.841–0.962
MSD Artec® Space Spider	0.914	0.824–0.958
MSD Bellus3D® Dental Pro	0.911	0.819–0.957
HD Planmeca® ProFace	0.999	0.997–0.999
HD Artec® Space Spider	0.984	0,967–0.992

ICC = Intraclass Correlation Coefficient; CI = Confidence Intervall; RMSE = Root Mean Squared Error; MSD = Mean Surface Distance; HD = Hausdorff Distance

**Table 3:** Intraclass correlation (ICC) to analyze the *intrarater* reliability of measurements performed by the two independent raters applying a two-way mixed model

Parameter	Rater 1 (CC)	Rater 2 (PK)
	*ICC (95%CI)*	*ICC (95% CI)*
**RMSE Planmeca® ProFace**	0.999 (0.998–1.000)	0.999 (0.997–1.000)
**RMSE Artec® Space Spider**	0.999 (0.997–1.000)	0.987 (0.963–0.995)
**RMSE Bellus3D® Dental Pro**	0.999 (0.996–1.000)	0.993 (0.981–0.998)
**MSD Planmeca® ProFace**	0.968 (0.908–0.989)	0.900 (0.716–0.965)
**MSD Artec® Space Spider**	0.995 (0.986–0.998)	0.808 (0.443–0.933)
**MSD Bellus3D® Dental Pro**	0.941 (0.829–0.979)	0.859 (0.591–0.951)
**HD Planmeca® ProFace**	0.998 (0.995–0.999)	0.999 (0.998–1.000)
**HD Artec® Space Spider**	0.999 (0.997–1.000)	0.969 (0.914–0.989)
**HD Bellus3D® Dental Pro**	0.999 (0.996–1.000)	0.983 (0.952–0.994)

ICC = Intraclass Correlation Coefficient; CI = Confidence Intervall; RMSE = Root Mean Squared Error; MSD = Mean Surface Distance; HD = Hausdorff Distance

### Root mean square error

**Fig. 4:**
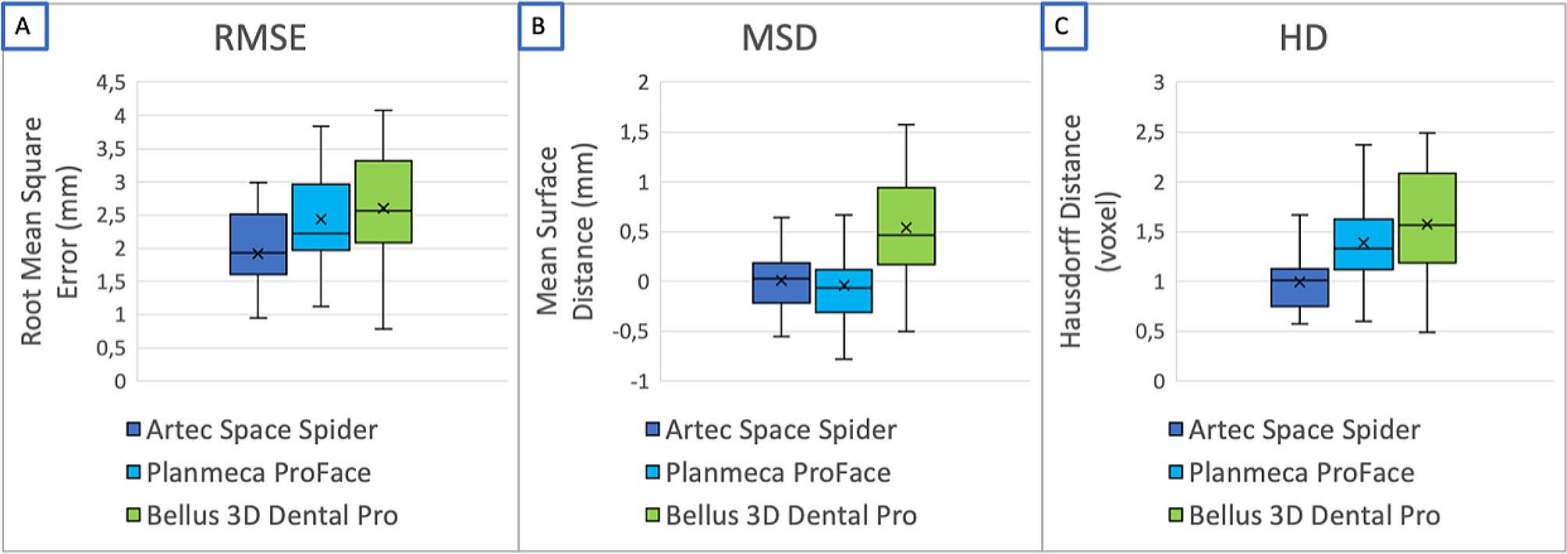
Three-dimensional analyses done with the open-source software MeshLab® showing **A:** The median root mean square error (RMSE, [mm]) **B:** The median mean surface distance (MSD, [mm]) **C:** The median of the Hausdorff distance (HD, [voxel])

The Artec Space Spider photography system showed the lowest RMSE (median 1.93 mm, range: 0.95–2.99; Fig. 4.) The one-factor analysis of variance (ANOVA) showed that the selection of the 3D-photography system had a significant effect on the RMSE (F = 15.136, *p* < 0.001, ηp2 = 0.138, *n* = 16). The effect size was f = 0.41, corresponding to a strong effect according to Cohen ^15^. A Bonferroni post hoc test showed that not all 3D-photography systems differed significantly (*p* = 0.621). However, the Artec Space Spider 3D-photography system was significantly different from the other two systems in terms of RMSE (*p* < 0.001).

#### Mean surface distance

The MSD of the Bellus3D Dental Pro application had the highest deviation from the ground truth model with a median of 0.46 mm (range: -1.23–1.57) (Fig. 4). The one-factor ANOVA showed that the choice of 3D-photography system had a statistically significant effect on MSD (F = 15.770, *p* < 0.001, ηp2 = 0.143, *n* = 16). The effect size was f = 0.38 and corresponded to a medium effect according to Cohen ^15^. The Bonferroni post hoc test showed that the Bellus3D Dental Pro application differed significantly (*p* < 0.001) from the other two systems. There was no statistically significant difference between Planmeca ProFace® and Artec Space Spider in terms of MSD analysis (*p* = 0.766).

### Hausdorff distance

Artec Space Spider had the lowest HD with a median of 1.01 voxel (range: 0.57–1.67) (Fig. 4). The one-factor ANOVA showed that the choice of 3D-photography system had a statistically significant effect on HD (F = 27.416, *p* < 0.001, ηp2 = 0.225, *n* = 16). The effect size was f = 0.54, consistent with a strong effect according to Cohen ^15^. The Bonferroni post hoc test showed that in terms of HD, analogous to RMSE, the Artec Space Spider 3D-photography system was significantly different from the other two 3D-photography systems (*p* < 0.001). No significant difference in HD was found between the Planmeca ProFace® 3D-photography system and the Bellus3D Dental Pro application (*p* = 0.076).

### Linear regression analysis for confounding factors

Neither gender nor age had a statistically significant effect on RMSE, MSD, or HD (*p* > 0.05) (Table 4).

**Table 4:** Uni- and multivariable linear regression model of the virtual-postoperative RMSE, MSD, and HD results and possible confounding factors

Univariante linear Regression Analysis						
***Parameter***	***Root Mean Square Error***		***Mean Surface Distance***		***Hausdorff Distance***	
	***p***-value	95% CI	***p***-value	95% CI	***p***-value	95% CI
Gender	0.132	-0.407–0.054	0.058	-0.268–0.004	0.146	-0.256–0.038
Age	0.569	-0.032–0.017	0.537	-0.019–0.010	0.981	-0.016– 0.016
Ethnicity	0.663	-0.376-0.590	0.506	-0.444-0.220	0.503	-0.211-0.429

### Surface distance map

**Fig. 5:**
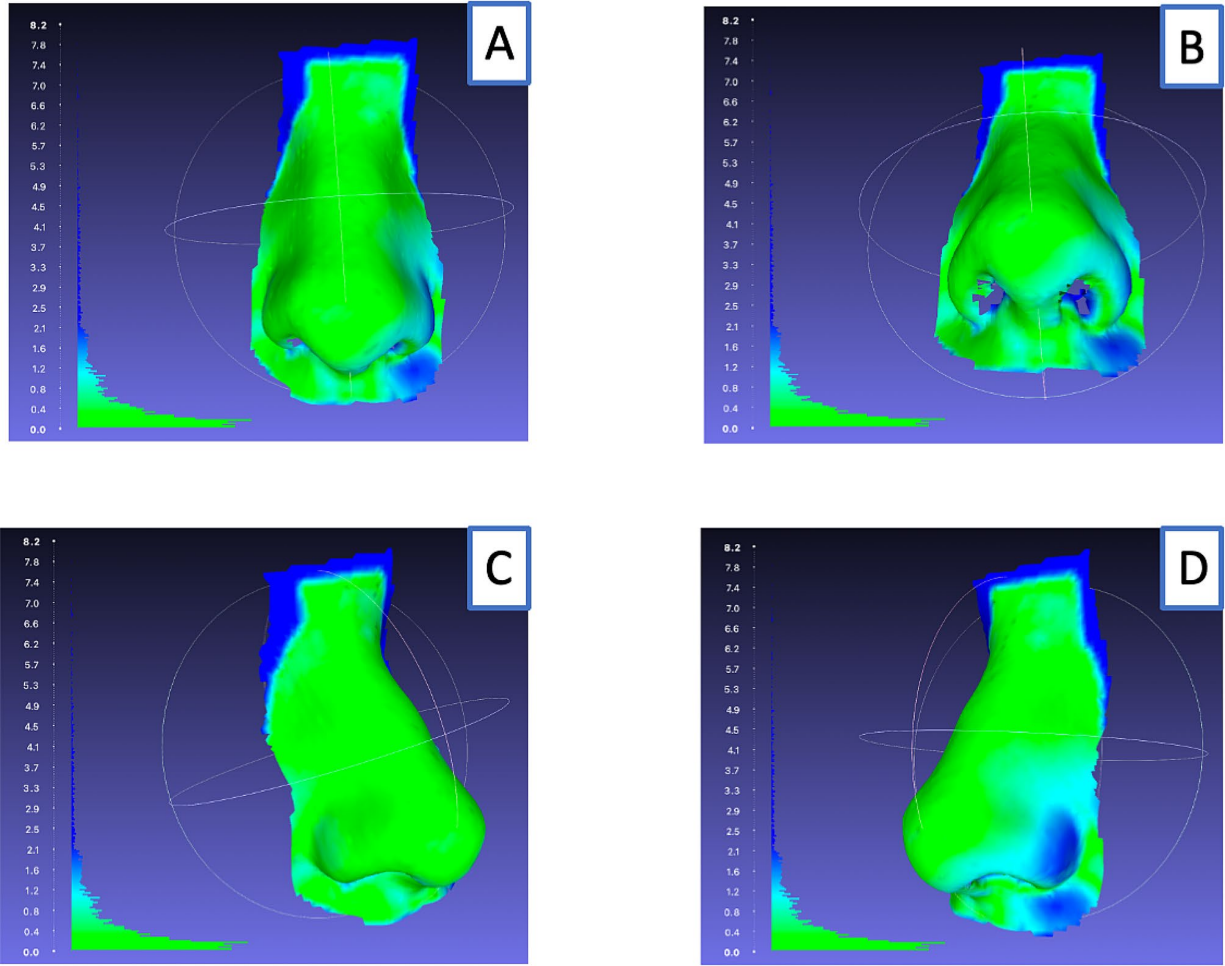
Surface distance map of Artec Space Spider of subject 7 **A:** Frontal view **B:** Basal view **C:** Side view right **D:** Side view left

The surface distance map for the Artec Space Spider 3D-photography system showed a very good agreement overall (Fig. 5). The upper marginal areas in the nasal root region showed the greatest distance. A side-by-side comparison showed that the left nostril had a lower match than the right.

## Discussion

In this study, we evaluated the accuracy, precision, and feasibility of three different 3D-photography systems. Facial 3D photography has reached a high level of accuracy and reproducibility, even with portable devices [18, 19]. The results showed that the Artec Space Spider system had the lowest RMSE and the smallest MSD among the three systems tested. This is consistent with previous studies that have also found the Artec system to have high accuracy and reliability [8, 20]. The Bellus3D Dental Pro application had the highest deviation from the ground truth model (MSD = 0.46 mm), which is in line with some previous studies that have reported limitations with this system [21]. Thurzo et al.‘s study compared the differences between the facial surfaces from CBCT and the Bellus3D Dental Pro application and described that the face scans deviated by more than 3 mm in some facial regions, which limits clinical use in orthodontic applications [22]. However, it should be noted that other studies have reported good accuracy with the Bellus3D system and they also stated that this system may provide 3D models of the face with clinically acceptable precision and reliable tools for planning surgical procedures [23, 24]. Results of other studies also confirm that the TrueDepth sensor used with Bellus 3D Dental Pro application provides sufficiently accurate data to design and print viable extraoral parts of orthodontic appliances in patients with craniofacial disorders [25]. Clinical studies indicate that a discrepancy in MSD of up to 2 mm for 3D facial photography is clinically acceptable [26–28]. Differences in the study designs, alignment algorithm, and sample characteristics could account for the divergent findings between studies [11, 29, 30]. By collecting all datasets of a test person at the same time, an influence on the part of the test person, such as weight gain or loss, hormonal, or time-of-day influences, could be excluded. However, the influence on the part of the test person by minimal mimic movements and breathing were included in the investigation. The influence of minimal movement during data collection could potentially explain the observed deviations in the measurements of the nostrils. These subtle facial movements can affect the positioning and shape of the nostrils, leading to slight differences in the recorded parameters. In comparison to investigations on static models such as a doll’s head [27, 31], this type of data collection corresponds more realistically to clinical examination conditions. Our study also found good interrater and intrarater reliability for all three parameters and for all three 3D-photography systems. This is consistent with previous studies using the same analysis algorithm [32].

Since the algorithm tries to achieve the smallest possible distance between the surface meshes during superimposition, it is possible that anatomical misclassifications occur. This source of error cannot be detected and mathematically represented using the global parameters RMSE, MSD, and HD. This problem with the use of the ICP algorithm was also shown in the study by Marliere et al. [30]. Only the color-coded surface distance map provided visual information about the anatomical mapping and allowed additional verification of the anatomical correctness of the superimpositions. Our results show that the color-coded surface distance map is conclusive with the parameters RMSE, MSD, and HD.

It is worth noting that there are several factors that can affect the accuracy of 3D-photography systems, including lighting conditions, camera resolution, and surface texture of the object being scanned [19, 33]. Therefore, it is important to consider these factors when choosing a 3D-photography system for a specific application. The results of our study indicate that different 3D-photography systems have varying suitability for specific clinical applications. For applications requiring the highest precision and accuracy, such as studying post-surgery facial swelling or volume changes after filler application, the Artec Space Spider system was found to be suitable. Despite its high acquisition costs (approximately €20,000), this portable device offers excellent precision. However, the ongoing costs for the annual software updates of approximately €2,000 should not be overlooked. Another aspect of the Artec Space Spider camera that must be taken into consideration is that despite its high precision and accuracy, its application is not exclusive to the medical field. This has the effect that the data are often processed in another program and thus further software licenses are necessary.

On the other hand, the Planmeca ProFace® system is particularly well-suited for soft tissue analysis or simulation in orthognathic patients. Its integration with the existing cone beam device allows for fast acquisition and easy overlay with cone beam images taken simultaneously. This makes it convenient for orthognathic surgery planning and assessment. When purchasing a new cone beam device for clinics with a high number of orthognathic surgical cases, it is worth the additional investment of approximately €4,000 for the ProFace® option. No additional software is required for the processing of the data.

For a more affordable (€40 per month) and mobile option, the Bellus3D application shows promise in everyday clinical use. It provides a simple, objective, and reproducible evaluation of soft tissue changes in terms of shape, volume, and symmetry. This makes it useful for individual planning and documentation in procedures like septorhinoplasty, mandibular reconstruction, orthognathic adjustment, and progress monitoring in cleft lip and palate patients. However, despite the detailed accuracy of the systems, the problem of simulating exact structural post-operative changes remains. The respective artificial intelligence models are still in testing phases and seem to play a central role in the future application [34, 35]. When discussing the cost-effectiveness of these 3D-photography systems it is important to analyze not only the initial purchase price but also factors like software maintenance, storage, and potential long-term value in medical or dental applications.

In conclusion, our study adds to the growing body of literature on the accuracy and reliability of 3D-photography systems. While the Artec Space Spider system showed the best accuracy in our study, it is important to consider the specific requirements of a given application when choosing a 3D-photography system. Future research should continue to explore the potential of 3D-photography systems for various clinical and research applications. Future efforts should focus on extending the capabilities of 3D photography to other regions of the face and body and for applications in 3D printing to fabricate customized appliances for patients.

### Limitations

There are several limitations to this study that should be acknowledged. First, the sample size may appear relatively small, which could limit the generalizability of our results. A sample size calculation was performed before recruiting subjects. The estimated effect size was 1.33 with an α-error probability of t = 0.05, power: 0.95. The result of the minimum number of cases was eight. This shows that the selected number of cases with sufficiently good power can prove the results. Second, we only evaluated the use of 3D photography in the context of the anatomical region of the nose and did not assess its applicability in other areas of plastic and reconstructive surgery. Further we attempted to control for factors such as lighting and patient positioning, there may be other confounding factors such as the alignment algorithm that we did not account for that could affect the mathematical results of the accuracy and precision measurements. Likewise, the alignment algorithm in MeshLab. When both meshes are not of equal size, it can result in areas that cannot be properly matched or assigned. This problem is particularly evident in the marginal regions, which are not yet in the area of most interest.

Finally, the Bellus 3D Dental Pro application ceased being available for purchase since December 1st, 2021 but the reason for discontinuation of the product remains unclear. As an alternative for Bellus3D Dental Pro, the literature discusses several mobile and app solutions, including Capture, Heges, and Scandy, which utilize monoscopic photogrammetry and LiDAR technology [36]. The discontinuation of Bellus3D does not render iPhone-based 3D scans unavailable. When comparing app-based solutions using an iPhone, it is essential to differentiate between apps using the front camera with True Depth Technology and apps using the back camera including the LIDAR scanner. It is evident that keeping abreast of app-based solutions can be challenging, given that such solutions are often initiated by start-ups with vague survival timelines.

## Conclusions

The three 3D-photography systems evaluated in this study have theoretical applicability in clinical routine for imaging the anatomical region of the nose. The results confirmed statistically significant differences between the different 3D-photography systems. While Artec Space Spider showed the highest accuracy, the Bellus3D Dental Pro app as a representative for iPhone X TrueDepth-based app solutions may be the most feasible option for everyday clinical use due to its portability, ease of use, and low cost. However, the specific purpose should be considered when selecting a 3D-photography system for clinical use. For applications demanding utmost precision and accuracy, such as operative planning, analyzing post-surgery facial swelling or evaluating volume changes after filler application, the Artec Space Spider system seems the most suitable of the investigated devices. In contrast, the Planmeca ProFace® system proved to be particularly advantageous for soft tissue analysis and simulation in orthognathic patients. Its integration with cone beam imaging allows for efficient acquisition and overlay, facilitating orthognathic surgery planning and assessment. For more everyday clinical use, the iPhone X TrueDepth-based app solutions showed promise in providing a simple, objective, and reproducible evaluation of soft tissue changes, making it suitable for individual planning and documentation in a variety of procedures.

## Data Availability

The datasets used and analyzed during the current study are available from the corresponding author on reasonable request.

## Abbreviations

3D: Three dimensional
ANOVA: Analysis of Variance
CBCT: Cone beam computed tomography
HD: Hausdorff distance
ICC: Intraclass correlation
ICP: Iterative Closest Point
LED: Light-emitting diode
MSD: Mean surface distance
RMSE: Root mean square error
STL: Standard tessellation language
